# Effective Relationships Between Younger Caregivers and Older Care Recipients Across a Continuum of Formal Residential Care Settings: A Scoping Review and a Critical Analysis

**DOI:** 10.3389/phrs.2024.1606562

**Published:** 2024-03-27

**Authors:** Carol-Ann Dixon, Vera Roos, Matthews Katjene, Jaco Hoffman

**Affiliations:** ^1^ Optentia Research Unit, North West University, Vanderbijlpark, South Africa; ^2^ Oxford Institute of Population Ageing, University of Oxford, Oxford, United Kingdom

**Keywords:** effective relationships, formal residential care, older persons, caregivers, relational care

## Abstract

**Objective:** This article aims to conduct a scoping review of what constitutes effective relational interactions between caregivers (CGs) and older persons (OPs) across formal residential care settings.

**Methods:** A scoping review of publications between January 2000 and December 2021 yielded 10,929 articles, and after removing duplicates and applying exclusion criteria, 36 articles were analysed.

**Results:** Articles were scrutinised for interactions involving both CGs and OPs, using a thematic framework analysis to identify effective relational constructs. Four themes emerged: 1) Diverse perspectives on the same context: for OPs it is home, and for CGs, workplace. 2) CGs move for a one-up position and OPs submit to a one-down, or as friends. 3) Relational qualities have been mostly associated with CGs, confirming care as a unidirectional action 4). Relationships between CGs and OPs result either in effective or ineffective care outcomes.

**Conclusion:** The dual meanings attached to the same context limit the authentic interactions between CGs and OPs. We propose a relational caregiving approach by considering the interactions of both CGs and OPs, changing the relational definition, and demonstrating effective relational qualities.

## Introduction

This article focuses on caregiving across formal residential care settings as a context to develop transferable relational principles applicable within any organised formal care setting. Formal residential settings offer care across the spectrum of independent living, assisted living to full-time frail care. The increase in future cohorts of OPs [1–3], presents an urgent challenge to better understanding OPs’ care needs. Even though the care of OPs is viewed primarily as a family concern [4, 5], families may be faced with limited financial and time resources, changing family demographics, and sometimes strained interpersonal relationships [1, 6–9], which necessitates some level of organised support. Formal care provided by paid professionals or carers focuses on physical care, nursing, or psycho-medical interventions [10]. To counteract the perceived ‘dehumanising’ approach to care associated with a medical model, person-centred care (PCC) approaches developed [11–14], such as: the Eden Alternative, which considers OPs’ emotional, social, and psychological needs while promoting their autonomy and self‐determination [15, 16]; the Values, Individualised, Perspective and Social (VIPS) model, which endorses the individual values and needs of OPs; Green Houses, advocating for the autonomy and dignity of OPs; Dementia Care Mapping, which focuses on the needs of OPs with dementia [17]; the Senses Framework emphasising the interdependence between OP and CG [18]; and, Swanson’s middle-range theory of caring explaining the process whereby caring is enacted [19].

Drawing on an ethic of care philosophy [20], PCC highlights the context in which caregiving takes place, and the quality of the relationship involving both CGs and OPs [15, 21, 22]. Research clearly associates effective relationships with healthy ageing [23, 24], but to date, research on the nature of relationships between OPs and CGs in formal care reports ineffective relationships [7, 17]; or focuses (punctuates) on the intrapersonal level (the subjective experiences) [7, 14, 17]. Scoping reviews deal with the quality of care for older adults [14]; life-story work in long-term care of OPs [25]; the perceptions and experiences of PCC from the CGs’ perspective [17]; and care-related research [7]. What remains underexplored is: What are the constructs underpinning effective relationships involving CGs and OPs in formal residential care contexts? Relationships, drawing on the interactional approach are the interactions (observable verbal and nonverbal messages) involving both CGs and OPs. Every interaction has an impact (a registering effect) on the recipient and a corresponding reaction, which in turn impacts the sender [26–29]. Moreover, interactions between people are always embedded in a particular environment, which informs behaviour [28]. The article aims to identify constructs underpinning effective relationships involving both CGs and OPs across a continuum of formal residential care contexts in the extant literature.

## Methods

### Information Sources and Search Strategy

Arksey and O’Malley’s [30] five stages guided the scoping review [1]: identify the research question(s) [2]; identify relevant studies [3]; data extraction [4]; chart the data; and [5] collate, summarise, and report the results. The PRISMA-P (Preferred Reporting Items for Systematic Review and Meta-Analysis Protocols) [31] checklist was used to ensure rigour. Scoping reviews involve an iterative and rigorous process; hence this protocol was revised as needed [32].

The following databases were searched: EBSCOHost (Academic Search Premier, Africa-Wide Information, Ejournals, ERIC, PsycArticles, PsycINFO, and SocINDEX), Scopus, African Journals (previously SAePublications) and Web of Science. The search strategy was based on the methodology and guidance of Peters et al. [33]. The full list of Boolean phrases used during the search is presented in Supplementary Table S1 (see Supplementary Material). The eligibility criteria applied included: publications in English between January 2000—December 2021; including both CGs and OPs (aged 60+) across a continuum of residential care settings; studies including OPs who are cognitively intact; and studies presenting interactional dynamics involving both CGs and OPs. The run-up to the Madrid International Plan of Action on Ageing (MIPAA) in 2002 and the subsequent momentum of ageing research broadly motivated the focus of 2 decades following that dynamic. Studies excluded were: ineffective relationships (e.g., elder abuse), studies with children or youth samples, informal or kinship carers, OPs in hospital settings, medically trained healthcare personnel, nursing for specific medical conditions (e.g., HIV, Alzheimer’s), and studies including OPs with dementia or cognitive impairment. Publication types not accepted for this review were literature reviews, book reviews, policy documents, government documents, grey literature, and non-peer-reviewed studies (e.g., training manuals, reference to blogs, newspaper articles or magazine articles).

**FIGURE 1:**
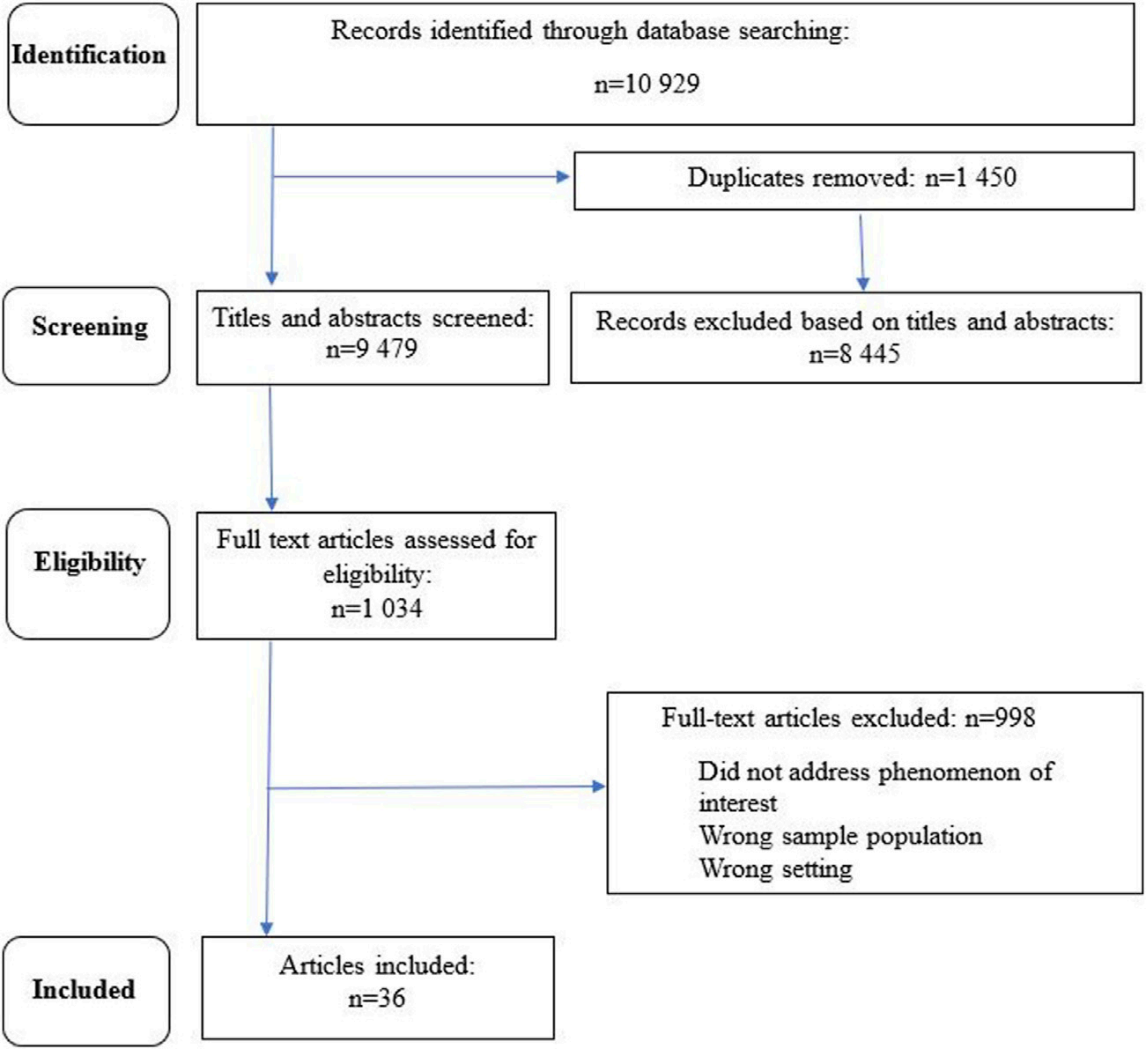
Flow diagram illustrating scoping review process (Worldwide, 2000–2021).

During the search process, three co-researchers independently identified 10,929 articles and screened article titles, abstracts, and keywords for eligibility. After duplicates had been removed and exclusion and inclusion criteria applied, 1,034 articles were read, disagreements resolved with consensus discussions, and 36 papers analysed. The PRISMA-P process is presented in Figure 1.

Data were charted in the following categories: preliminary details: date of study, author/s, journal source, article title, aim of study, sample, research design, data-collection method, and findings (see Supplementary Material), following Arksey and O’Malley [30] and Peters et al. [33]. Contextual details included were: country, research setting, and participants’ perspectives. The software program Atlas.ti22 was used to assign preliminary codes, which were discussed and reviewed to create categories, and refined into themes.

## Results

### Description of Included Studies

The selected literature is generally skewed towards the feminisation of care and Global North contexts: North America (n = 11), Europe, Scandinavia and United Kingdom (n = 16), New Zealand (n = 1), South America (n = 1), Middle East (n = 2), Asia (n = 3), and Africa (n = 2), presented in Supplementary Table S2 (see Supplementary Material). Country classifications according to developed or developing are based on the United Nations World Economic Situation and Prospects [34]. The socio-economic context plays a role in care resources, but here the focus is to identify effective relational constructs transferable across contexts.

Articles included report the perspective of OPs regarding care perceptions and experiences [35–40]; care for thriving [41]; emotional containment [42], dignity [43, 44]; trust [45]; and OPs’ experiences of their relationship with CGs [27, 46–48]. From the perspective of CGs, studies include the meanings associated with the role of caregiving [49–53]; communication strategies in caregiving [54–56]; and the competence of CGs [57, 58]. Studies focusing on the care relationship both from OPs’ and CGs’ perspectives include the requirements to perform caregiving [59]; the meaning of care [60–62]; the effects of ethnic differences [63]; beliefs about and assumptions about care [64]; and the social needs of OPs [65]. A few studies explored the care relationship in long-term care from multiple perspectives, including family members and staff [66–69].

The articles focused mostly on CGs while the relational qualities of OPs are seldom reported, implying that caregiving is a unidirectional act without consideration of the complex interplay between OPs and CGs [35, 46, 48, 60, 67, 68]. Studies punctuating on the relational level, describe heuristic constructs of social closeness and distance [63] and the concept of reciprocity [48, 51, 56, 67] as important in relational dynamics. The literature is, however, vague on practical application, and lacks a nuanced discussion of effective relational constructs. While the concept of reciprocity is reported as a mutual exchange in relationships between OPs and CGs [48, 51, 56, 66, 67] the focus does not adequately describe relational dynamics between both CGs and OPs (and in most cases only focus on one party). The quotations in the studies yielded rich descriptive data about the interactions between OPs and CGs, which were critically analysed using a thematic analysis framework to identify effective relational constructs.

### Relational Constructs Informing Interactional Dynamics

**FIGURE 2:**
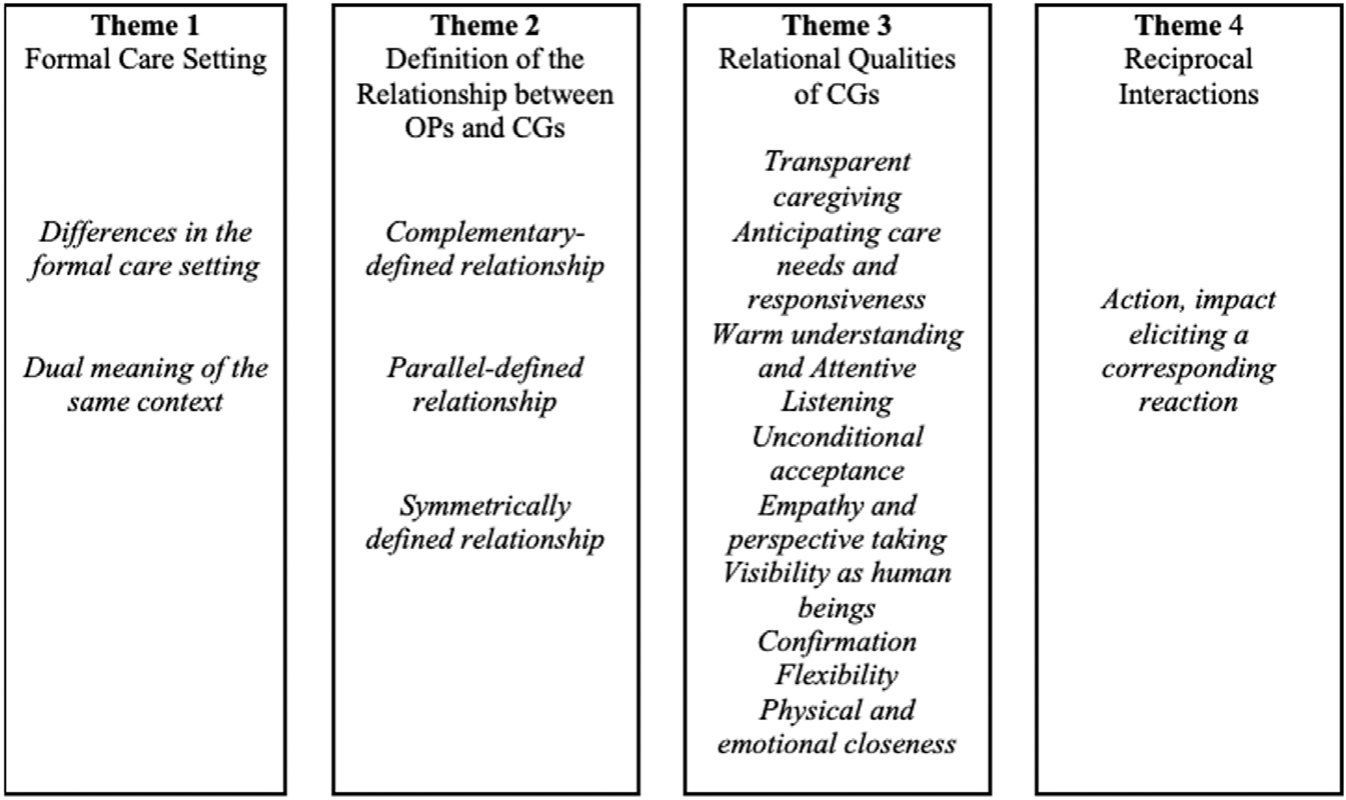
Themes and subthemes relevant to relational interactions between older persons and caregivers (Worldwide, 2000–2021).

Four themes and 15 subthemes are presented in Figure 2.

### Formal Care Settings

#### Diverse Perspectives in the Formal Care Setting

Relational interactions between CGs and OPs take place in a formal care environment characterised by diversity: age, socio-cultural norms, language, and ethnicity. Age differences are mentioned in relation to younger migrant care workers. Socio-cultural differences manifest in the daily practices, for example, when CGs are serving tea to the OPs. In the example below, the OP experienced frustration in relation to CGs who did not act according to the OP’s expected socio-cultural norms:

How to make a cup of tea, for example, she [Hungarian migrant care worker] was just making half a cup of milk and just a bit of tea. So, everybody was criticising the tea because it was cold, and I taught her how to make it (62, p.17).

Language differences impact differently on OPs and CGs. OPs experienced the language barrier as inhibiting their spontaneous reactions, such as using humour: “You know, we do not understand [them], and you cannot always joke because they do not understand the joke” (OP) (62, p.16). CGs try to deal with the language differences by consulting a dictionary, but this impacts the flow of the interaction with OPs: “It’s like we were talking to the residents with the dictionary and the patients were just waiting you know while you check with the book” (CG) (62, p.16). On the other hand, when OPs and CGs share a common language, the relationship deepens: “The nurses take his hand, and they know a lot of words in Russian. He is looked after well. He kisses the hand of the nurses and calls them sweetheart” (CG) (67, p. 4).

Age and ethnic differences between OPs and CGs led to the assignment of stereotypical labels. CGs, for example, reported as follows: “I keep thinking, ‘Oh, they’re old or they’re senile’” (CG) (64, p.116). When OPs and CGs interact effectively, they challenge stereotypical labelling and adopt new behaviour, as illustrated in the following example from the perspective of a CG:

One lady, she looked at me and says: “Oh, you’re a colored girl.” And I said, “What’s that?” And she said, “You know, colored.” And I said, “No, I do not know what that is. You’re going to have to tell me what that is.” She says, “You know, you people are called colored people.” And I said, “No, I think we’re called African American.” And I said, “Like you, you’re probably labelled as European American.” And she says, “Is that what they’re doing now?” I said, “Yes.” She goes, “Well, tell me that again, so I can remember it.” So she does not use the word “colored” anymore, she says “African American” now (64, p.118).

#### Ambiguities of Expectations in the Same Context

The context is simultaneously the workplace of the CGs while also the home of the OPs, as a CG explains: “Often we have to remember that we’re working in their home, they’re not living in our workplace” (CG) (58, p. 150). Context informs behaviour, illustrated by the use of time. CGs are required to perform tasks according to a set schedule: “Everything is time, …. and if you do not do it in a certain way then you run out of time and then you end up rushing and not giving quality care” (51, p. 2). When some OPs feel the pressure of time in their interactions with CGs, they do not want to cooperate: “If she is giving me a bath and she is rushing, I would not want to take a bath. If she has the time to sit and talk with me, it makes me feel that I can trust her” (OP) (36, p. 8).

### Definition of Relationship Involving OP and CG

In every interaction, individuals move for control, which is explained by how the relationship is defined such as: one individual adopts a one-up position and the other accepts the control by adopting a one-down position (complementary), a relationship between equals where control is flexibly shared between interacting individuals (parallel); and, a non-acceptance of the control by either individual leading to escalations, misunderstanding, and frustration (symmetrical) [29, 70, 71]. The relationship between CGs and OPs mostly emerged as complementary, with CGs in the one-up position and OP in a one-down position: “The staff would sit in the shared living room for their coffee breaks and led the conversation with the residents” (OP) (48, p. 7). However, when CGs and OPs interact as friends or equals (parallel-defined relationship), they share information mutually: “She does not see me anymore as a patient, she sees the person behind that. I tell her about myself, and she tells me about herself” (OP) (36, p. 7). This type of relational definition enables OPs and CGs to become more visible as human beings, sharing jokes and enjoying the relationship as indicated below:

Both of us undressed and stood there in our bras and then we started to laugh … here—you and I, she said, in our bras! (laughing) and we’re going to try on blouses (CG) (45, p. 7).

In the residential setting, the assumption is that CGs are leading the caregiving relationship. However, when OPs demonstrate keen interest and involvement in the direction and quality of their own care, a symmetrically-defined relationship is observed illustrated by the following example:

I’m really used to directing my own care … and that does not always go over real well with people in a place like this because I’m used to telling people what to do and having it done when I tell them to do it (OP) (68, p. 64).

### Relational Qualities of CGs

From the perspective of both OPs and CGs, CGs should have the following relational qualities: transparent caregiving, anticipating OPs’ needs, warm understanding, unconditional acceptance, empathy, perspective taking, visibility as a human being, confirmation, flexibility, physical and emotional closeness.

**Transparent caregiving** refers to the notion that CGs should be clear in terms of what the act of care involves and what is expected of OPs, illustrated in the following quote: “They tell me what they are going to do, and I like it” (OP) (61, p. 7).

In **anticipating OPs’ care needs** CGs apply their professional experiences and knowledge of interpersonal behaviour to respond appropriately, expressed by a CG: “You know the noise … you know the gestures they make you … You already know before they ask you … you already know what they mean what they want” (CG) (61, p 6). CGs get to know OPs’ needs by spending time with them: “Coming to work every day … I walk around a lot … and I know what they want” (CG) (61, p. 6). Responding appropriately means that CGs attend to the OP’s needs promptly. CGs’ quick and consistently reliable responses contribute to a sense of interpersonal safety, according to an OP: “I always say I feel safe with her. If I ask her for something, she does it … and you know that she will come back with somebody very soon” (OP) (36, p.7). When OPs experience interpersonal safety, they engage more freely with CGs: “… eventually they’ll feel safe enough to come out, but you just got a make it happy otherwise it’s not going to get that way” (CG) (57, p. 47).

**Warm and understanding** CGs provide an inviting interpersonal space, as illustrated by an OP, who said: “I choose the person who willingly listens to my problems and is able to give good advice” (OP) (43, p. 8). CGs who are warm and listen attentively to OPs and repeat (or mirror) what the OP has said are regarded as reliable. An OP expressed it as follows: “If she (CG) has the time to sit and talk with me it makes me feel that I can trust her. That is important to have someone who you can trust” (36, p.8).

**Unconditional acceptance** means a total and authentic acceptance of another person, without judgement. This is described by the OP: “Yes, she calms me down and she really takes me as I am, she is a wonderful person” (41, p. 4).

**Emotional empathy** means that the CG is attuned to the emotional needs of the OP and responds appropriately, illustrated below:

I saw that the resident could not stop crying in her bed when I was walking around the room, so I asked what had happened … She told me something about her family history … I comforted her patiently … she felt a lot better … Since then, she always tells me that I am the one who really cares about her (the resident) … She gave me fruit occasionally … This showed me that if you care about the residents, they appreciate that (CG) (65, p. 1).

**Taking the perspective** of the OPs elicit empathy in CGs: “I guess I was just thinking at that moment about him and how he felt … if that had been me, you know? What would I have wanted someone to do to make me feel more better about the situation?” (CG) (50, p. 8). When CGs display empathy and adopt the perspective of the OP, the OP responds with cooperative behaviour:

Well, put yourself in their shoes. If you were laying there all night and somebody walks in with their, “Oh, we’ve got to get you up.” Would you want to get up? Would you want that person to even touch you? So walk in and say good morning, put a smile on your face and cheer them up and get them in a good mood that they would want to get out of bed (CG) (51, p. 1).

OPs confirm that the experience of perspective taking and empathy from CGs results in an outcome of trust: “Residents thought that empathy involving an understanding of their situation, perspective, and feelings was a necessary basis for constructive and trusting relationships with staff” (36, p. 7).

**Being visible as a human being**, means people are authentic or truly themselves. When OPs and CGs act authentically, differences seem to diminish: “We usually joke. It feels good … we’re the same … was not different from her and she was not different from me (giggling)” (CG) (45, p. 7). Becoming more visible in the relationship is also promoted through a prolonged relationship [72]. One of the outcomes of treating OPs as human beings is that they become actively engaged in their own care [50] because they feel taken seriously, and not categorised as a patient.

**Confirmation** refers to acknowledging the other and can take different forms, for example, when CGs greet OPs: “Good morning is a big thing for them. I think greeting is more of psychological reassurance that had value in their life” (CG) (66, p. 8). Confirmation also means validating OPs’ experiences and their needs: “Caring for the whole person includes treating residents like individuals, viewing the patient as a person, responding to emotional undertones or needs and validating the resident experience, wishes and values” (CG) (51, p. 1). OPs also confirm CGs, illustrated in the following description by a CG: “You feel really confident in your job, and you feel important for them” (61, p.13). Eliciting a reaction from being confirmed is illustrated in the following non-verbal message from an OP: “Seeing the result … a smile possibly, from someone who has been low, and suddenly it’s there … this positive aspect of one’s efforts, when one succeeds, it is like seeing life being restored” (CG) (53, p. 4).

**Being flexible** means adapting to another’s needs or behaviour. In this instance a CG describes how she responds to OPs: “They all have different preferences, and you have to adjust and you have to do what they prefer, because you are taking care of them” (CG) (51, p. 1). Being flexible in a caregiving context means that care is situational and specific to the individual relationship.

**Closeness** in a relationship is experienced on a continuum of being too close or too distant and can be expressed **physically or emotionally**. Physical closeness expressed by touch creates safety for an OP, who may need reassurance and support: “Like just touching their hand or holding their hand and giving them security in a different way” (CG) (51, p.1). Physical closeness is also expressed when a CG is a comforting companion for an OP and joins in what they want to do: “Or when a resident who sits next to me and wants to watch TV, so I go with her and we watch together the programme; like being her mother, being close” (CG) (60, p. 5). Emotional closeness involves sharing emotions, losses, pain, laughter, and joy: “We have shared very intimate experiences. This brings you close together, so many tragedies and so many good times … and they are the ones standing next to you” (OP) (49, p. 554).

Three relational outcomes emerged from emotional closeness both for CGs and OPs. First, OPs become attached: “I am becoming very attached to them and I love them, just love them as my own grandchildren” (OP) (36, p. 7). Second, the relationship contributes to greater visibility: “We are like granddaughter and granny, the relationship is like that. We always have a good laugh, we always talk about everything” (CG) (62, p. 12). Third, it transgresses differences: “The nurses take his hand, and they know a lot of words in Russian. He kisses the hand of the nurses and calls them sweetheart” (67, p. 4). The disruption of a physically and emotionally close relationship creates a deep sense of loss, as illustrated by the following example:

There is this pretty young girl … I like her as I like my grandchildren and she treats me not just like a patient but she sees me like her grandma. She knows intimate details about me, and she knows my likes and dislikes. But now they transferred her to another unit. I truly miss her (OP) (36, p. 9).

### Reciprocal Interactions

Reciprocal interactions mean that both OPs and CGs are involved in the interaction (consisting of **action, impact, and a corresponding reaction**) [26, 28]. This reciprocity in the interaction is reported by CGs as well as OPs, as illustrated in the following two examples: “A conversation would be more meaningful if both parties are listening to each other attentively. You will listen to me; I will listen to you” (OP) (43, p. 8); and “You will talk about you and your family, and they will talk about them and their family and their husbands, and then give you tips that you do not know” (OP) (61, p. 5). One CG describes the behaviour she might elicit from an OP as a result of her actions: “They’ll (OP) respond to you positively if you treat them right. If you do not, they are not like a light bulb. You just cannot switch them off … If you have been rude, they remember how you treated them. If you are nice to them, they will know it” (CG) (50, p. 6).

## Discussion

The sample sizes in the 36 qualitative studies were generally small (30 study participants or fewer) and only two studies included sample sizes of more than 80 CGs and OPs [52, 61]. The search strategy was challenging because the construct caregiver was used inconsistently and interchangeably with nurses, nurse-aides, care managers, and other healthcare workers [18, 19, 27, 51–53, 57–59, 62, 63, 66, 69]. The exclusion criteria for OPs did not distinguish between mild and severe cognitive impairment, and as a result some studies could have been excluded. It is recommended that future studies include the voices of this target group. Despite the inclusion of both OPs and CGs, studies focused more on CGs’ effective relational qualities and very little on OPs’ relational qualities, and interactions.

### Theoretical Analysis

Analytical approaches to understanding the relational dynamics between CGs and OPs in formal care settings are presented in Supplementary Table S3 (see Supplementary Material), with one article [37] using two theoretical lenses.

Intrapersonal approaches, theoretical frameworks and heuristic constructs explain experiences of relationships from within a person; how CGs and OPs make sense and understand the meaning of and in care [40, 49, 53, 62, 64], OPs self-determination [37], affect containment [42], CGs’ care philosophy and motivation to care [39, 50, 52], CGs’ cultural competence and relational qualities [57–59] and OPs needs [36, 38, 41, 43–45, 65]. These frameworks and heuristic constructs provided explanations on an intrapersonal level. Interpersonal theories that explain interactions between people include communication theories [35, 46, 54, 55], and theories describing a good fit between CG environment and OPs [37, 47, 61, 69]. Theories describing relational interactions [18, 19, 43, 47, 60, 61, 68, 69] do not adequately explore OPs’ observable responses to caregiving.

Potential relational dynamics can be extracted from three articles, not primarily developed from a healthcare perspective: Buber’s I-Thou using the construct of mutuality [68], but it fails to explain observable interactions between CGs and OPs. Relational Dialectics Theory [60] assumes three tensions in relationships: connectedness vs. separateness, certainty vs. uncertainty, and openness vs. closedness but lacks an explanation of effective relationships between CGs and OPs. The Self-Interactional Group Theory (SIGT) explains relational interactions and the dynamic interplay on three levels: intrapersonal, interpersonal, and group [27]. The intrapersonal presents emotions and perceptions as the impact informing what is taking place on the interpersonal and group levels. The interpersonal level analyses the interaction from five perspectives: context, definition of the relationship, relational qualities, motivation for the interaction, and the circular processes of interaction. The group level includes group dynamics within and between groups. SIGT suggests that relational dynamics are embedded in specific environments [73]. The nature of the formal caregiving context creates a dissonance: CGs are performing their care duties, and interacting with OPs in an unidirectional way while OPs accept the care as recipients. CGs are in the one-up and OPs in a one-down position. In this position, OPs may choose to resign their autonomy (and parts of who they are), even though they may be able to care for themselves [47, 65]. In this caregiving context, OPs may not always be viewed as active participants in the relationship.

### Limitations

When interpreting the results, it should be kept in mind that drawing exclusively on English publications may have eliminated useful research. Caregivers encompass a broad range of people who do not interact directly with OPs in a reciprocal relationship. The inconsistent definitions of caregiving and the selection of specific search engines could have presented biased results. However, this first known scoping review presents detailed knowledge of what constitutes effective relational constructs between OPs and CGs in formal residential care environments.

### Conclusion

This scoping of articles and data focuses on the relationships between CGs and OPs across a continuum of formal residential care contexts. Results indicated that the formal caregiving context unintentionally creates dissonance in the relationship involving OPs and CGs (e.g., home vs. workplace; manoeuvres for control in the relationship). In this sense, both CGs and OPs redefine their autonomy, and care itself becomes the ultimate goal, thereby repositioning the relational context and content. Despite the assumption that PCC care in formal care settings adopts a relational focus, the context is inherently counterproductive to the development of authentic human relationships. The relational qualities underpinning effective relationships highlight the unidirectional nature of care and relating. We therefore recommend adopting a relational caregiving approach considering the interactions of both CGs and OPs; a (re)-designing of how the formal care contexts function by intentionally focusing on the co-creation of authentic (equal) relationships. To this end, the care should be the outcome of effective relationships and not the goal. This study also identified the need for an integrated conceptual framework to analyse the interactions between CGs and OPs in formal care settings toward developing effective relationships.
