# Supplementary material for: Effective Relationships Between Younger Caregivers and Older Care Recipients Across a Continuum of Formal Residential Care Settings: A Scoping Review and a Critical Analysis
Source: Public Health Rev. 2024 Mar 27;45:1606562. doi: 10.3389/phrs.2024.1606562 (PMC11004294; doi:10.3389/phrs.2024.1606562)
Supplement: Supplementary file 2 [file Table1.DOCX]

**Table 1.** Boolean Phrases to identify articles regarding the relationship between caregivers and older care recipients in a formal care context

|  | **Search Strings** | Hits  (before excl. criteria) | After exclusion criteria | Total included after reading full article |
| --- | --- | --- | --- | --- |
| 1 | (Care-giver* OR Carer OR “Elder care” OR  “Non-kinship care” OR “Formal carer” “Formal care*” OR nurse OR Allied health personnel OR “Care provider”) AND (“Care receiver” OR “Care recipient” OR patient) AND (Elder* OR “Old* people”) AND (“Old age home” OR “Care home” OR “Residential care”) AND (relation* OR interaction* OR “social closeness”) | 1336 | 318 | 13 |
| 2 | (Care-giver* OR “Non-kinship care” OR “Formal carer” “Formal care*”) AND (patient OR “gerontological community”) AND (Aged OR pensioner*) AND (Elder* OR “old* people” OR geriatric) AND (“Assisted living“ OR Nursing homes OR “Retirement home”) AND (“Interactional patterns” OR “Relational dynamics" OR relation* OR interaction* OR Connection OR Communication OR “Social closeness” OR Reciproc* OR Interpersonal relations OR Intergenerational relations) | 225 | 20 | 0 |
| 3 | (Care-giver* OR Carer OR “Elder care” OR “Non-kinship care” OR “Formal carer” “Formal care*” OR nurse OR Allied health personnel OR “Care provider”) AND (“Care receiver” OR “Care recipient” OR patient OR “gerontological community”) AND (“Old age home” OR “Care home” OR “Residential care”) AND (relation* OR interaction*) | 2938 | 156 | 6 |
| 4 | (Care-giver* OR “Non-kinship care” OR “Formal carer” “Formal care*” OR Allied health personnel OR “Care provider”) AND (patient OR “gerontological community”) AND (“Nursing homes" OR “Retirement home”) AND (relation* OR interaction* OR reciproc* OR communication) | 610 | 60 | 0 |
| 5 | (Care-giver* OR Carer OR “Elder care” OR “Non-kinship care” OR “Formal carer” OR nurse OR Allied health personnel OR “Care provider”) AND (“Care receiver” OR “Care recipient” OR patient OR “gerontological community”) AND (“Retirement facility” OR respite care OR Homes for the aged) AND (relation* OR interaction*) | 1997 | 94 | 7 |
| 6 | (Care-giver* OR Carer OR “Elder care” OR “Non-kinship care” OR “Formal carer” “Formal care*” OR nurse OR Allied health personnel OR “Care provider”) AND (“Care receiver” OR “Care recipient” OR patient OR “gerontological community”) AND (Assisted living facility* OR “Long-term care” OR “private care homes”) AND “Social closeness” OR Reciproc* OR “Interpersonal relations” OR “Intergenerational relations” | 235 | 40 | 10 |
| 7 | (Care-giver* OR Carer OR “Elder care” OR “Non-kinship care” OR “Formal carer” “Formal care*” OR nurse OR Allied health personnel OR “Care provider”) AND (“Care receiver” OR “Care recipient” OR patient OR “gerontological community”) AND (“congregate housing” OR “Community based care”) | 236 | 86 | 0 |
| 8 | (Care-giver* OR “Elder care” OR “Non-kinship care” OR “Formal carer” “Formal care*” OR Allied health personnel) AND (Elder* OR “Old* people”) AND (“Interactional patterns” OR interaction*) | 1641 | 138 | 0 |
| 9 | (Care-giver* OR “Elder care” OR “Non-kinship care” OR “Formal carer” “Formal care*” OR Allied health personnel) AND (Elder* OR “Old* people) AND ("old age home" OR "retirement facility" OR "assisted living facility" OR "long-term care") AND (connection OR communication OR relation*) | 1293 | 73 | 0 |
| 10 | (Care-giver* OR Carer OR “Elder care” OR “Non-kinship care” OR “Formal carer” “Formal care*” OR nurse OR Allied health personnel OR “Care provider”) AND (“Care receiver” OR “Care recipient” OR patient OR “gerontological community”) AND (“Old age home” OR “Care home” OR “Residential care”) AND (relation* OR interaction*) | 1388 | 49 | 0 |
|  | Total | 10929 |  |  |
