# Supplementary material for: Effective Relationships Between Younger Caregivers and Older Care Recipients Across a Continuum of Formal Residential Care Settings: A Scoping Review and a Critical Analysis
Source: Public Health Rev. 2024 Mar 27;45:1606562. doi: 10.3389/phrs.2024.1606562 (PMC11004294; doi:10.3389/phrs.2024.1606562)
Supplement: Supplementary file 3 [file Table3.DOCX]

**Table 3.** Theories and heuristic constructs

| **References** | Theoretical approaches, theories and heuristic constructs | Summary of approach |
| --- | --- | --- |
| ***Intrapersonal approaches*** (not theories) explaining experiences of relationship from within a person | | |
| 41. Westin, L., & Danielson, E. (2007). Encounters in Swedish nursing homes: a hermeneutic study of residents' experiences. Journal of advanced nursing, 60(2), 172–180. | Meaning in life defined as being somebody versus being nobody | Explores OPs’ feelings about their encounters with CGs: Being somebody versus being nobody; Being part of a community, Existing as a person and the impact this has on the meaning in their lives. |
| 50. Anderson, R. A., Ammarell, N., Bailey, D., Jr., Colón-Emeric, C., Corazzini, K. N., Lillie, M., Piven, M. L. S., Utley-Smith, Q., & McDaniel, R. R., Jr. (2005). Nurse Assistant Mental Models, Sensemaking, Care Actions, and Consequences for Nursing Home Residents. Qualitative health research, 15(8), 1006-1021. | Mental models  Sense making | A mental model is an explanation or representation of a person’s inner thoughts, which may motivate behaviour and decisions.  Sensemaking is how people make sense of their environment to make decisions.  CGs use mental models and sensemaking to make sense of OPs needs and how this impacts their caregiving. |
| 54. Funk, L. M., & Outcalt, L. (2020). Maintaining the ‘caring self’ and working relationships: A critically informed analysis of meaning-construction among paid companions in long-term residential care. Ageing & Society, 40(7), 1511-1528. | Meaning making  The meaning of care | CGs’ meaning of care and the caring self. |
| 63. Figueredo-Borda, N., & Zabalegui-Yárnoz, A. (2015). Long-Term Care of the Elderly in Uruguay. Journal of Transcultural Nursing, 26(2), 178-184. | Meaning making  The meaning of care | Meaning of care, experience of care, continuity of care for the fulfilment of OPs’ needs. |
| 65. Lung, C.-C., & Liu, J. Y. W. (2016). How the perspectives of nursing assistants and frail elderly residents on their daily interaction in nursing homes affect their interaction: a qualitative study. BMC geriatrics, 16, 13. | Intention and beliefs | Intentions or beliefs of CGs and residents direct their interactive behaviour. |
| ***Intrapersonal theories*** explaining experiences of relationship from within a person | | |
| 38. Custers, A. F. J., Westerhof, G. J., Kuin, Y., Gerritsen, D. L., & Riksen-Walraven, J. M. (2012). Relatedness, autonomy, and competence in the caring relationship: The perspective of nursing home residents. Journal of Aging Studies, 26(3), 319-326 | Self-determination theory | Self-determination theory: three universal basic psychological needs, important for psychological growth and well-being.  1. relatedness (connected and belonging)  2. autonomy  3. competence.  Need fulfilment provided by relationship with CGs related to OPs well-being. |
| 40. Hwang, H. L., Hsieh, P. F., & Wang, H. H. (2013). Taiwanese long-term care facility residents' experiences of caring: a qualitative study. Scandinavian Journal of Caring Sciences, 27(3), 695-703 | Swanson’s Caring process theory | The core philosophy of the Swanson’s Caring Process theory is that humans cannot be treated as objects and cannot be separated from self, other, nature, and the larger workforce. How CGs enable OPs to achieve well-being and fulfil their needs. |
| 43. de Guzman, A. B., Valdez, L. P., Pascasio, B. N. D. C., Pascual, F. J. C., & Pelayo, S. C. (2017). When everything is under control: Chronicling the affective containment experiences of Filipino elderly in institutionalized settings. Educational Gerontology, 43(5), 226-237. | Affect containment theory | OPs contain, regulate or project emotions to cope in response to stress  How OPs cope in Long Term Care (LTC). |
| 51. Eaton, J., Cloyes, K., Paulsen, B., Madden, C., & Ellington, L. (2020). Certified nursing assistants as agents of creative caregiving in long‐term care. International Journal of Older People Nursing, 15(1). | Structural empowerment theory | Structural Empowerment Theory – If a person believes they have influence, they feel empowered.  If CGs perceive they have an influence on job (on OPs or management), more likely to feel empowered and use creative caring behaviours. |
| 53. Eldh, A. C., van der Zijpp, T., McMullan, C., McCormack, B., Seers, K., & Rycroft‐Malone, J. (2016). ‘I have the world's best job'—Staff experience of the advantages of caring for older people. Scandinavian Journal of Caring Sciences, 30(2), 365-373. | Eriksson’s  caritative  theory | Love as a central aspect and motivation in caring in order to fulfil OPs’ needs |
| ***Heuristic constructs* (**part of a theory) explaining experiences of relationship from within a person | | |
| 37. Bowers, B. J., Fibich, B., & Jacobson, N. (2001). Care-as-service, case-as-relating, care-as-comfort: Understanding nursing home residents' definitions of quality. The Gerontologist, 41(4), 539-545. | Autonomy and  social relationships | Care-as-relating in findings: references to importance of individualised care, choice and autonomy, reciprocity and social relationships to fulfil OPs need for quality care. |
| 39. Dybvik, T. K., Gjengedal, E., & Lykkeslet, E. (2014). At the mercy of others - for better or worse. Scandinavian Journal of Caring Sciences, 28(3), 537-543. | Dependency | OPs’ experiences of dependency in LTC. |
| 42. Bergland, Å., & Kirkevold, M. (2005). Resident-Caregiver Relationships and Thriving among Nursing Home Residents. Research in nursing & health, 28(5), 365-375. | Thriving | Thriving: The ongoing process of interaction with environment, resulting in social, physical, and psychological resilience and growth. |
| 45. Holmberg, B., Hellström, I., Norberg, A., & Österlind, J. (2019). Assenting to exposedness – meanings of receiving assisted bodily care in a nursing home as narrated by older persons. Scandinavian Journal of Caring Sciences, 33(4), 868-877. | Self-determination versus  dependency | Receiving assisted bodily care (R-ABC) creates tension between autonomy of OP and dependency on care. |
| 46. Stuck, R. E., & Rogers, W. A. (2019). Supporting trust in home healthcare providers: Insights into the care recipients’ perspective. Home Health Care Services Quarterly, 38(2), 61-79. | Trust and  vulnerability | OPs’ trust in CGs measured by their willingness to be vulnerable. Trust is what allows OPs to receive care despite the potential risks associated with doing so. How CGs engender trust in OPs. |
| 58. Tayab, A., & Narushima, M. (2015). “Here for the Residents”: A Case Study of Cultural Competence of Personal Support Workers in a Long-Term Care Home. Journal of Transcultural Nursing, 26(2), 146-156. | Cultural  competence | Cultural competence is the ability to understand different cultures and adapt accordingly, needed to meet needs of OPs. |
| 59. Villa, G., Pennestrì, F., Rosa, D., Giannetta, N., Sala, R., Mordacci, R., & Manara, D. F. (2021). Moral Distress in Community and Hospital Settings for the Care of Elderly People. A Grounded Theory Qualitative Study. Healthcare (Basel, Switzerland), 9(10). | Moral Distress  CGs’ relational qualities | CGs’ experiences of Moral Distress (MD). Caregiving and relational qualities needed to deal with MD when working with OPs. |
| 60. Band‐Winterstein, T., Doron, I., Zisberg, L., Shulyaev, K., & Zisberg, A. (2019). The meanings of the unlicensed assistive personnel role in nursing homes: A triadic job analysis perspective [Article]. Journal of Nursing Management (John Wiley & Sons, Inc.), 27(3), 575-583. | Characteristics for  good care | CGs’ personal qualities for good care for fulfilment of OPs’ needs. |
| 66. Teka, A., & Adamek, M. E. (2014). “We prefer greeting rather than eating:” Life in an elder care center in Ethiopia. Journal of cross-cultural gerontology, 29, 389-404. | Dependency syndrome  Caregiving relationship defined as home | Dependency syndrome defined as an attitude and belief that a group cannot solve its own problems without outside help.  OP’s perceptions of caregiving relationship as “home”. |
| ***Heuristic constructs*** explaining interactions between people | | |
| 44. Høy, B., Lillestø, B., Slettebø, Å., Sæteren, B., Heggestad, A. K. T., Caspari, S., Aasgaard, T., Lohne, V., Rehnsfeldt, A., Råholm, M.-B., Lindwall, L., & Nåden, D. (2016). Maintaining dignity in vulnerability: A qualitative study of the residents’ perspective on dignity in nursing homes. International Journal of Nursing Studies, 60, 91-98. | Dignity-in-  Relation  Confirmation | OPs internal experience of dignity maintained when confirmed by CGs when they reflect worth and value back to the individual through word or deed. |
| 49. Palacios-Ceña, D., Losa-Iglesias, M. E., Gómez-Calero, C., Cachón-Pérez, J. M., Brea-Rivero, M., & Fernández-de-las-Peñas, C. (2014). A qualitative study of the relationships between residents and nursing homes nurses. Journal of Clinical Nursing, 23(3-4), 550-559. | Reciprocity in terms of OPs creating balance in relationship | Mutual exchange between two individuals in a relationship. Caregiving relationship unbalanced, with CGs giving care. OPs use various strategies to create balance and reciprocity. |
| 52. Edvardsson, J. D., Sandman, P. O., & Rasmussen, B. H. (2003). Meanings of giving touch in the care of older patients: becoming a valuable person and professional. Journal of clinical nursing, 12(4), 601–609. | Reciprocity through meaningful touch | Touch as a vehicle for relational connection between CGs and OPs. |
| 57. Medvene, L. J., & Lann-Wolcott, H. (2010). An exploratory study of nurse aides communication behaviours: Giving positive regard as a strategy. International Journal of Older People Nursing, 5(1), 41-50. | Reciprocity and positive regard as relational strategies | Positive regard and reciprocity are strategies which takes a caregiving relationship beyond task orientation. |
| 64. Berdes, C., & Eckert, J. M. (2001). Race relations and caregiving relationships: A qualitative examination of perspectives from residents and nurse's aides in three nursing homes. Research on aging, 23(1), 109-126. | Distance and closeness in  relationships | Ethnic differences in facilities create social distance between residents and staff. |
| 67. McGilton, K. S., & Boscart, V. M. (2007). Close care provider–resident relationships in long-term care environments. Journal of Clinical Nursing, 16(11), 2149-2157. | Reciprocity defines close relationships | Concept of “close relationship” defined and examined from CGs, OPs and family perspectives. Close relationships achieved through reciprocity between OPs and CGs. |
| 68. Roberts, T., & Bowers, B. (2015). How nursing home residents develop relationships with peers and staff: A grounded theory study. International journal of nursing studies, 52(1), 57-67. | Reciprocity in terms of human responsiveness | Back and forth interaction depending on the elicited reaction.  OPs strategies in developing relationships with CGs depend on responsiveness of CGs as positive or negative. |
| ***Interpersonal theories*** explaining interactions between people | | |
| 36. Adra, M., Aharonian, Z., & Sibai, A. M. (2019). Exploring resident-staff relationships in nursing homes in Lebanon. International Journal of Qualitative Studies on Health & Well-Being, 14(1), 1-12. | 7 Cs  Continuity of  care | 7 Cs: the factors necessary to promote “appreciative caring conversations” for compassionate care.  Continuity of care relies on  CGs characteristics: reliability, empathy to fulfil OP needs. |
| 38. Custers, A. F. J., Westerhof, G. J., Kuin, Y., Gerritsen, D. L., & Riksen-Walraven, J. M. (2012). Relatedness, autonomy, and competence in the caring relationship: The perspective of nursing home residents. Journal of Aging Studies, 26(3), 319-326. | Person-Environment  (P-E) Congruence Model | Person-Environment (P-E) congruence model: a person's well-being will be affected by the fit between their important needs and the environment around them.  Need fulfilment provided by relationship with CGs related to OPs well-being. |
| 47. Marsden, S., & Holmes, J. (2014). Talking to the elderly in New Zealand residential care settings. Journal of Pragmatics, 64, 17-34. | Communication  accommodation theory (CAT) | CAT seeks to explain when, how, and why individuals adjust verbal and non-verbal interactions with others, as well as recipients’ inferences, attributions, and evaluations of, and responses to, them in order to fulfil OP needs. |
| 48. Nakrem, S., Vinsnes, A. G., & Seim, A. (2011). Residents’ experiences of interpersonal factors in nursing home care: A qualitative study. International Journal of Nursing Studies, 48(11), 1357-1366. | Senses Framework from CGs frame of reference | The Senses Framework -  the interpersonal relationship between resident and CGs implies long-term commitment, reciprocal relationships and understanding of each resident’s needs. |
| 55. Jansson, G., Wadensjö, C., & Plejert, C. (2017). Managing complaints in multilingual care encounters. Multilingua, 36(3), 313-345. | Communication  Analysis | How CGs communicate with OPs to maintain an intimate and cheerful atmosphere. How they deal with complaints and fulfil their needs. |
| 56. Plejert, C., Jansson, G., & Yazdanpanah, M. (2014). Response practices in multilingual interaction with an older persian woman in a swedish residential home. Journal of Cross-Cultural Gerontology, 29(1), 1–23. | Communication  Analysis | The systematic examination of verbal and nonverbal behaviours to understand how people communicate with each other and maintain interactional social order. Cultural and linguistic matching between CG and OP important for fulfilment of OP needs. |
| 62. Walsh, K., & Shutes, I. (2013). Care relationships, quality of care and migrant workers caring for older people. Ageing & Society, 33(3), 393-420. | Senses Framework from both CGs and OPs frame of reference | Senses Framework used to understand CG’s (migrant) and OP’s perceptions of quality of care, relational aspects of good care. Focus on reciprocity and discrimination. Influence on care delivery and organisational structure. |
| 70. Wilson, C. B., & Davies, S. (2009). Developing relationships in long term care environments: the contribution of staff. Journal of Clinical Nursing (Wiley-Blackwell), 18(12), 1746-1755. | Nolan’s Senses Framework from CGs frame of reference | Focus on education of caregivers to include understanding of 6 senses.  Relationship qualities  of CGs.  Relationship-centred care  defined as care for OPs,  negotiated with all people  in OP’s life.  How enabling relationships are developed and the relationship qualities needed for creating relationship-centred care approach to care of OPs with the outcome of a positive experience of care. |
| ***Interpersonal theories*** explaining relational dynamics between people | | |
| 28. Roos, V., & Du Toit, F. (2014). Perceptions of effective relationships in an institutional care setting for older people. *SA Journal of Industrial Psychology, 40*(1). | Self-Interactional Group theory | SIGT explains the relational/interactional nature of intergenerational relations. Relationships are viewed as the reciprocal, continuous communicative interactions between members of different generations. |
| 61. Levy-Storms, L., Claver, M., Gutierrez, V. F., & Curry, L. (2011). Individualized care in practice: Communication strategies of nursing aides and residents in nursing homes. Journal of Applied Communication Research, 39(3), 271-289. | Relational dialectic theory | Relational dialectics theory assumes three different types of tensions in relationships: connectedness vs. separateness, certainty vs. uncertainty, and openness vs. closedness. |
| 69. Westerhof, G. J., van Vuuren, M., Brummans, B. H. J. M., & Custers, A. F. J. (2014). A Buberian approach to the co-construction of relationships between professional caregivers and residents in nursing homes. The Gerontologist, 54(3), 354-362. | Buber I-Thou  mutual co-construction of relationships | Buber (1958/2000) distinguished between I-Thou and I-It relationships. I-It is uni-directional, I-Thou is bi-directional. The relationship between the CG and the OP is co-constructed and quality of care is mutually constructed. |
